# Circular from the Berlin Polyclinic

**Published:** 1884-08

**Authors:** Ludwig Loewe

**Affiliations:** Surgeon-General of the Berlin Polyclinic; Berlin


BERLIN, 13 June, 1884.

*Dear Sir:*—The Berlin Polyclinic begs you, in the interest of those medical gentlemen anxious for an opportunity of going through a course of training in special subjects, kindly to copy the following in the editorial columns of your valuable paper.

Clinical courses, comprising all the different special branches for practical physicians, are held every month in the Polyclinic at Berlin (Germany), Carlstrasse 30. The courses always commence on the first week day of the month. They last a whole month and are held every working day.

The number of participants is limited to six for every course. Should more than six apply for the same course, an extra—or parallel—course will be formed.

To all those physicians wishing to perfect themselves in a special branch, the opportunity is given to serve three months as assistants in that particular branch. Those gentleman having served as assistants will be allowed in appropriate cases to conduct the extra or parallel courses.

We intend to elevate the Berlin Polyclinic to an international medical school for the improvement of physicians of every country. In order to have the courses conducted in foreign languages, assistantships will be conferred also to foreign physicians.

Yours, very respectfully,

DR. LUDWIG LOEWE,

Surgeon-General of the Berlin Polyclinic.

